# Dopamine modulation of sensory processing and adaptive behavior in flies

**DOI:** 10.1007/s00441-020-03371-x

**Published:** 2021-01-30

**Authors:** K. P. Siju, Jean-Francois De Backer, Ilona C. Grunwald Kadow

**Affiliations:** School of Life Sciences, Department of Molecular Life Sciences, Technical University of Munich, 85354 Freising, Germany

**Keywords:** Dopamine, Neuromodulation, Mushroom body, State-dependent behavior, Drosophila

## Abstract

Behavioral flexibility for appropriate action selection is an advantage when animals are faced with decisions that will determine their survival or death. In order to arrive at the right decision, animals evaluate information from their external environment, internal state, and past experiences. How these different signals are integrated and modulated in the brain, and how context- and state-dependent behavioral decisions are controlled are poorly understood questions. Studying the molecules that help convey and integrate such information in neural circuits is an important way to approach these questions. Many years of work in different model organisms have shown that dopamine is a critical neuromodulator for (reward based) associative learning. However, recent findings in vertebrates and invertebrates have demonstrated the complexity and heterogeneity of dopaminergic neuron populations and their functional implications in many adaptive behaviors important for survival. For example, dopaminergic neurons can integrate external sensory information, internal and behavioral states, and learned experience in the decision making circuitry. Several recent advances in methodologies and the availability of a synaptic level connectome of the whole-brain circuitry of *Drosophila melanogaster* make the fly an attractive system to study the roles of dopamine in decision making and state-dependent behavior. In particular, a learning and memory center—the mushroom body—is richly innervated by dopaminergic neurons that enable it to integrate multi-modal information according to state and context, and to modulate decision-making and behavior.

## Introduction

Making the right decision at the right time is very important for animal survival. These decision-making events are particularly crucial when animals are navigating their complex and dynamic environment for food, mates, breeding sites, or to escape from predators. In order to make decisions, for instance whether to turn, stop, or continue, animals usually evaluate signals from their current external environment and internal state, and integrate them with their past experiences or innate priors. As the environment and context changes and the internal needs fluctuate, animals are constantly making and updating decisions to adapt to both the environment and to their own behavioral state for survival. Because of this, every animal goes through decision-making processes many times a day and innumerable times in their life time. Most of the time, decisions have to be made instantaneously forcing animals to decide quickly and with a low margin of error. A flexible state-dependent decision-making ability thus provides essential adaptability to animals in their environment. Behavioral manifestations of decisions appear simple (e.g., eat, mate, escape, forage), yet the neural mechanisms by which state, context, and experience are integrated to enable flexible decision making involve complex and shared neural circuits in higher brain centers.

Neuromodulation is an excellent way to achieve neuronal and circuit flexibility—and thereby behavioral modification—at different levels and for different timescales by integrating multiple signals and reconfiguring circuits (Bargmann 2012; Bargmann and Marder 2013; Dayan 2012; Marder 2012). These modulations are enabled in animal nervous systems by several, evolutionarily conserved, neuromodulators including biogenic amines, neuropeptides, and neurotransmitters. They can work in concert or independently, locally or globally, and affect different synaptic properties of pre- or postsynaptic neurons to achieve neuromodulation (Marder 2012; Taghert and Nitabach 2012). Dopamine is one such molecule that has multiple, partially conserved, neuromodulatory functions in animal nervous systems (Scaplen and Kaun 2016; Watabe-Uchida and Uchida 2018). Dopamine is a ubiquitous molecule found in a wide spectrum of life forms from microorganisms to humans. It is a biogenic amine biosynthesized from tyrosine. In this biosynthetic pathway, tyrosine is first converted to the intermediate molecule L-DOPA by the action of the enzyme tyrosin hydroxylase (TH), and L-DOPA is further converted to dopamine by the enzyme dopa decarboxylase (DDC) (Budnik and White 1987). Dopaminergic neurons (DANs) synthesize, store, and release dopamine as synaptic transmission or volume transmission depending on the event. While synaptic transmissions are localized events only affecting the postsynaptic target neuron, volume transmission facilitates the release of dopamine into the circulating lymph, whereby it is transported to different parts of the brain (Rice et al. 2011). Therefore, dopamine can act fast and locally as well as slower and systemically. Dopamine is involved in the regulation of voluntary locomotion, learning and memory, and the neuroendocrine axis in several animal species including humans (Berke 2018; Bjorklund and Dunnett 2007; Bromberg-Martin et al. 2010; Coddington and Dudman 2019). In addition, dopamine is associated with motivation, need, and reward (Burke et al. 2014; Ito and Doya 2015; Schultz 2015; Watabe-Uchida and Uchida 2018). DANs encode behavioral and internal states of animals as well as sensory stimuli including odors (Aimon et al. 2019; Berry et al. 2015; Cohn et al. 2015; de Jong et al. 2019; Lewis et al. 2015; Lutas et al. 2019; Menegas et al. 2018; Riemensperger et al. 2005; Siju et al. 2020; Tsao et al. 2018). In particular, recent data strongly suggest that DANs integrate and convey the value of sensory information, current internal and behavioral state to appropriate decision-making centers in the brain, and thereby aid in forming and updating behavior and memory.

Due to a high degree of functional conservation, dopamine and its role in decision-making have been studied in many animal models, from primates to simpler invertebrates. While not appropriate for all aspects of studying decision-making and behavioral adaptation, *Drosophila melanogaster* along with the remarkable developments in methodologies to monitor, trace, and manipulate neurons and circuits (Owald et al. 2015b), has provided some key insights into the neuronal and neural circuit mechanisms of DANs and their role in higher brain centers (e.g., Aimon et al. 2019; Berry et al. 2015; Boto et al. 2019; Cohn et al. 2015; DasGupta et al. 2014; Felsenberg et al. 2017, 2018; Groschner et al. 2018; Ichinose et al. 2017; Kaun and Rothenfluh 2017; Lewis et al. 2015; Owald et al. 2015a; Owald and Waddell 2015; Riemensperger et al. 2013, 2005; Scaplen and Kaun 2016; Siju et al. 2020; Tomchik 2013; Watabe-Uchida and Uchida 2018; Yamagata et al. 2016)). In the following sections of this review, we provide an account of studies that discuss dopamine and its modulatory role in state-dependent behavior in flies*.* We aim at highlighting the diverse functions of brain dopamine in adult *Drosophila* by discussing recent, selected examples in the literature and what they could mean for research in higher animals. This review certainly does not cover all contributions to the field, but instead a selection of works with a particular focus to illustrate the diversity of functions of dopamine.

### Diverse roles and mechanisms of dopamine signaling

**Fig. 1:**
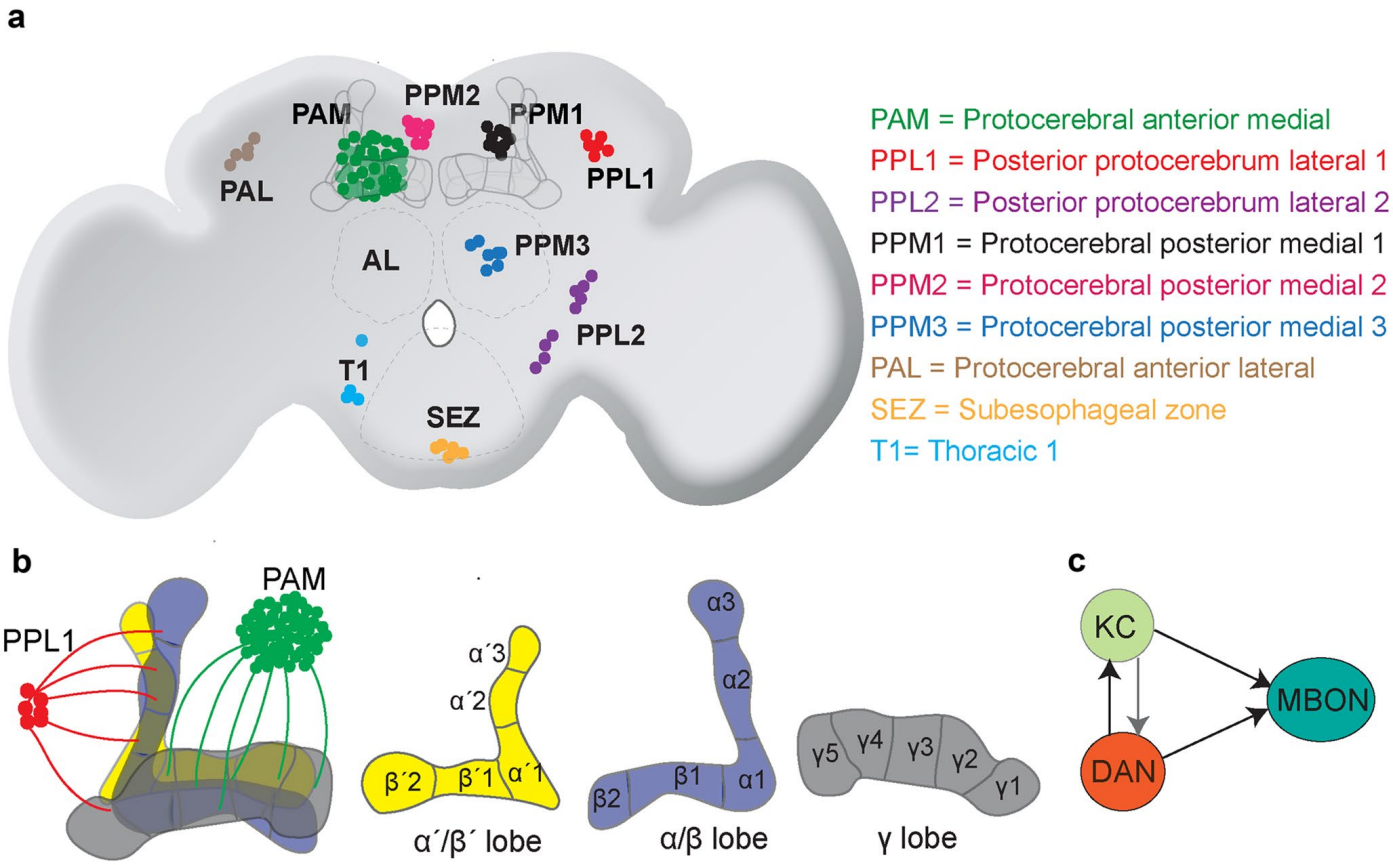
Overview of Drosophila dopaminergic neurons. (**a)** Schematic representation of the main clusters of dopaminergic neurons in the fly brain. AL antennal lobe. Adapted from Mao and Davis (2009) and Kasture et al. (2018). (**b)** Higher detail schematic of dopaminergic neurons innervating the mushroom body (MB). Left panel: PPL1 and PAM neurons innervate different compartments of the MB. Other panels: Schematic representation of different Kenyon cell (KC) axon bundles and their organization into different MB lobes. (**c)** Representation of a canonical recurrent module of a dopaminergic neuron (DAN), a KC and a MB output neurons (MBON)

Pioneering immunocytochemical studies have shown that the *Drosophila* brain contains around 130–140 DANs present in thirteen distinct clusters per hemisphere spread across different parts of the brain (Budnik and White 1988; Mao and Davis 2009; Nässel and Elekes 1992). DAN clusters are named according to the location of their cell bodies in the brain (Fig. 1a). The majority of these clusters are found in the protocerebral area and show connections to higher brain centers such as the mushroom body (MB) and central complex (CC), which are each involved in control and modulation of several behaviors (Azanchi et al. 2013; Kasture et al. 2018; Mao and Davis 2009).

A mammalian brain contains orders of magnitudes more dopamine-producing neurons. These are mainly found in the ventral tegmental area (VTA) and substantia nigra of the midbrain. DANs from these nuclei send projections mainly to the striatum but also to other brain areas (Bjorklund and Dunnett 2007). Despite their relatively small number as compared to other neuron types, the axon of a single DAN covers a large volume of tissue and can therefore modulate a large number of neurons in its target area (Matsuda et al. 2009). *Drosophila* DAN axons also densely innervate the MB, in a segregated organization similar to the compartmentalization of the striatum, the main target of DANs in vertebrates (Watabe-Uchida and Uchida 2018). Though it is unlikely that DAN clusters in insect brains can be directly compared with mammalian DAN-containing brain regions, the discrete organization and small overall number of DANs and the small size of the fly brain itself allows the study of complete DAN populations and even whole brain networks.

Importantly, DANs show a remarkable degree of conservation between different species in terms of their cellular biology. In vertebrates, DANs show a continuous, albeit low rate baseline activity known as tonic firing. By contrast, stimulus driven bursting DAN activity was termed a phasic response. Typically, in most of the DANs, the phasic response appears to work as a reward prediction error: DANs fire according to a difference between the actual and the predicted reward (Schultz et al. 1997). However, subsequent work showed that some DANs respond to aversive rather than rewarding stimuli (Matsumoto and Hikosaka 2009), leading to an updated “motivational salience” model where DANs signal the detection of an important stimulus for the animal, thereby promoting a behavioral reaction, and subsequently reinforcing it if and when appropriate (Bromberg-Martin et al. 2010). By now, several additional phasic signals have been reported; in particular, many DANs appear to respond to movement even in the absence of external stimuli (Dodson et al. 2016; Howe and Dombeck 2016; Schultz 2019).

The diversity of responses, cell types, and connectivity led to the proposition of the existence of multiple, heterogeneous DAN populations and systems acting in concert to shape the animal’s behavior (Watabe-Uchida and Uchida 2018). Transient phasic responses to various sensory stimuli and during behavior are now well established in flies (Cognigni et al. 2018; Ichinose et al. 2017). As in mammals, different subsets of DANs respond to attractive or aversive stimuli (e.g., Siju et al. 2020). Furthermore, many DANs across the brain are activated during walk (Aimon et al. 2019). Similar to mammals, a response in a particular fly DAN is elicited when something novel or unexpected occurs (Hattori et al. 2017). The response rapidly decays and disappears after repeated stimulations with this odor as it becomes familiar.

In vertebrates, tonic DAN activity supposedly maintains a steady low concentration of dopamine in the target areas, and may provide a basal firing line enabling the system to signal a negative prediction error or an aversive event between actions (Bromberg-Martin et al. 2010). In addition to these different firing patterns, recent work suggests an important contribution of local regulation of dopamine concentration by the striatum local microcircuit (Berke 2018; Cover et al. 2019; Mohebi et al. 2019; Threlfell et al. 2012). This might be responsible for slow increases of dopamine concomitant to approach behavior and motivation. For technical reasons, most of the neuronal activity recordings of DANs in the fly brain have been performed using calcium imaging. As this technique relies on a relative measurement and very few electrophysiological recordings have been published so far (Pimentel et al. 2016), it is difficult to be certain of whether fly DANs display the same tonic pacemaker activity as mammalian DANs (Ichinose et al. 2017). Nonetheless, some specific DANs show slow spontaneous calcium oscillations reflecting the internal state of the animal (Aimon et al. 2019; Berry 2015; Cohn et al 2015; Placais et al. 2012; Plaçais and Preat 2013; Siju et al. 2020). This pattern resembles the slow oscillatory firing observed in the VTA of anesthetized rats in synchronization with the prefrontal cortex, a region forming reciprocal connections with the VTA (Gao et al. 2007; Shi 2005). Thus, in how far the same categorizations or theories apply to mammalian and non-mammalian models is still unclear.

Dopamine exerts its effect through different dopamine specific receptors present on the target cells. Dopamine receptors are GPCRs segregated in two major classes, D1-like and D2-like, differing in their structure, pharmacology, and their coupling to intracellular signaling cascades (Beaulieu and Gainetdinov 2011). Classically, D1-like receptors have a low affinity for dopamine and are positively coupled to the enzyme adenylyl cyclase, while D2-like receptors have a high affinity for dopamine and are negatively coupled to adenylyl cyclase. Due to their different affinities for dopamine, D1-like receptors respond better to phasic dopamine release and D2-like receptors to tonic dopamine release. Using these different modes of signaling, dopamine receptors can modulate the synaptic strength between two connected neurons both locally at the pre- and postsynaptic side as well as whole neuronal excitability per se (Tritsch and Sabatini 2012). In flies, there are four types of dopamine receptors: D1-like dopamine receptor include Dop1R1 (Dumb) and Dop1R2 (Damb), and a D2-like receptor also called as Dop2R. In addition, *Drosophila* also express a non-canonical receptor called DopEcR (dopamine/ecdysteroid receptor). As in mammals, fly dopamine receptors participate in different forms of synaptic plasticity (Handler et al. 2019; Modi et al. 2020). For instance, in the fly MB, Dop1R1 and Dop1R2 have been implicated in pre-synaptic depression and potentiation and in memory acquisition and forgetting, respectively (Berry et al. 2012). Even if both receptors are coupled to the adenylyl cyclase and promote the production of cAMP, this activity is primarily important for Dop1R1 and synaptic depression (Himmelreich et al. 2017). Dop1R2 is preferentially coupled to the protein Gαq and promotes the release of calcium from internal stores, leading to synaptic potentiation (Himmelreich et al. 2017). However, in the context of sleep, Dop1R2 hyperpolarizes neurons in the dorsal FB and thereby reduces sleep (Pimentel et al. 2016).

Recent large-scale single-cell transcriptomics and receptor gene expression analysis showed that the same neuron can expresses multiple receptors, for example, Kenyon cells (KCs), principle cells of the MB, harbor two or more dopamine receptor types, and sometimes all four receptors are co-expressed within the same KC axon (Croset et al. 2018; Kondo et al. 2020). Direct functional evidence of co-expression of Dop1R1 and Dop1R2 has been shown for MBON- γ1pedc, where receptors are differentially activated during successive phases of a learning protocol (Pavlowsky et al. 2018). Similarly, differential Dop1R1 and R2 activity appears to be responsible for the opposite timing-dependent effect of dopamine during aversion and relief learning (Handler et al. 2019). Therefore, it is critical to note that the strength and efficiency of neuromodulation also depends on the distribution and expression of the receptors on the target cells (Marder 2012; Marder et al. 2014). Since dopamine can act through different types of dopamine receptors, identifying the receptors that are involved and their respective signaling mechanisms is important to understand dopamine-mediated neuromodulation.

## The role of dopamine in state-dependent behavior in *Drosophila*

The large majority of behaviors are expressed in a state-dependent manner. This state could be both internal state, e.g., metabolic state, and behavioral state, i.e., moving, resting, of an animal. Therefore, a state of an animal is not constant, but instead can change from one moment to the next, within seconds, minutes, hours, or days. Expressing appropriate state-specific behaviors is key for animal survival, for example risking one’s life for foraging only makes sense when in current or foreseeable need of food. This also explains why internal states have a fundamental impact on valence and value perception of external sensory information, and moreover, these perceptions are constantly updated according to ongoing experience and behavioral state. For instance, the value of a reward directly correlates with the animal’s need state and effort invested to obtain it (Berke 2018). Dopamine plays a key role in dynamically representing these different aspects. How exactly DANs fulfill this complex role is insufficiently understood, and fly research has helped to fill some of the gaps in our knowledge.

In flies as in many other animal species, the major internal states include the feeding state, the reproductive state, sleep and wake states, but also emotional states such as aggressiveness, which can be displayed by both males and females. How the nervous system, which ultimately controls behavior senses these metabolic, physiological or emotional states is not well understood. It was hypothesized, and experimental evidence is being gathered, that molecular signals including hormones, neuropeptides, produced by internal organs such as the gut, are being perceived by neurons in the brain. These neurons include DANs.

### Chemosensory processing and perception as a model for state-dependent behavior

Several state-specific behaviors are modulated by external chemosensory inputs. One of the most studied sensory systems in flies is the chemosensory system, which comprises both olfactory and gustatory systems. *Drosophila* has a well-developed olfactory system that consists of peripheral olfactory organs such as antenna and maxillary palp and a main taste organ, the proboscis (legs and wings are also serve as taste organs). Olfactory receptor neurons (ORNs) and gustatory receptor neurons (GRNs) with specific receptors present in the respective organs detect the odorants and tastants, respectively, and transmit neural information to the primary processing centers called antennal lobe (AL) and subesophageal zone (SEZ) in the brain (Scott 2018; Vosshall and Stocker 2007; Wilson 2013). Processed sensory information from the primary olfactory centers are carried by PNs to higher brain centers (Bates et al. 2020). Higher chemosensory brain centers consist of two main structures, the lateral horn (LH) and mushroom body (MB). The LH is considered to be a brain structure that controls innate behaviors (Dolan et al. 2018; Jefferis et al. 2007; Strutz et al. 2014), while the other higher brain center, MB, is involved behavioral adaptation, including learning and memory, and predominantly receives olfactory information (Aso et al. 2014; Heisenberg, 2003; Li et al. 2020a; Modi et al. 2020).

Taste-related motor programs are mostly processed directly at the SEZ. So far, only a few taste PNs to the higher brain areas have been identified (Kim et al. 2017, Li et al. 2020a, Scott 2018). In addition, a previous study showed that taste is also represented in the MB as sparse coding (Kirkhart and Scott 2015).

### Dopaminergic neurons of the mushroom body

As described above, several nuclei or clusters of DANs are found in insect brains (Fig. 1). These DANs innervate different areas of the protocerebrum, the CC, and the MB. DANs in the insect protocerebrum have, for example, been implicated in reproduction and sex-specific behaviors of flies (Kuo et al. 2015). Nevertheless, the by far best studied DANs innervate the insect MBs. In parallel to the many studies on the role of the MB in associative (olfactory) memory formation, several recent studies in *Drosophila*, involving high resolution anatomy, neuronal manipulation, and behavioral analysis have elucidated how MB guide decisions and adapt ongoing behavior (Modi et al. 2020). Drawing heavy parallels from vertebrate studies, the complexity in the circuitry, and convergence of information from different sensory modalities and internal states, the MB possesses all the features of an important state-dependent decision-making center.

**Fig. 2:**
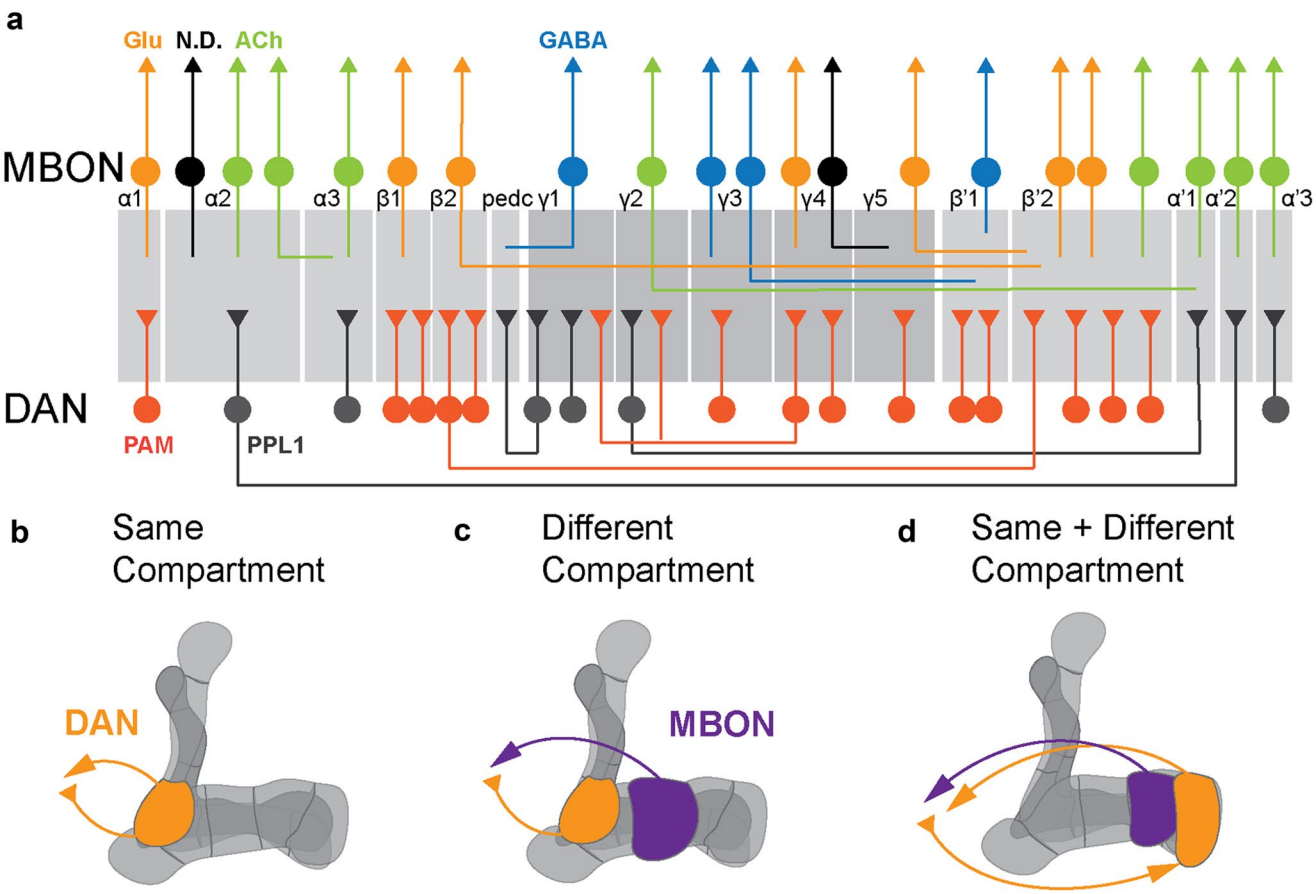
Mushroom body dopaminergic neurons form recurrent connections. (**a)** Simplified overview of different mushroom body (MB) compartments and their connections to dopaminergic neurons (DAN) and MB output neurons (MBON). Note that different compartments can be innervated by the same DAN type and same compartments can give output to multiple types of MBONs. Adapted from Li et al. (2020a). (**b)** DANs form recurrent synaptic connections with DANs within the same MB compartment. (**c)** DANs form recurrent connections with MBONs across different MB compartments. (**d)** DANs and MBONs can recurrently connect same and different MB compartments in parallel. Adapted from Li et al. (2020a)

Looking at the structural and functional organization of the *Drosophila* MB led to multiple comparisons to vertebrate brain centers in addition to the already mentioned basal ganglia. Recent development of genetic tools and high-resolution anatomical characterization, both at the light microscopic and electron microscopic level, showed a high level of cellular complexity, circuit connections, and computational power of the MB (Aso et al. 2014, Li et al. 2020b, Takemura et al. 2017, Zheng et al. 2018). One cannot help but notice the striking organizational similarities between the MB and the vertebrate cerebellum (Litwin-Kumar et al. 2017; Modi et al. 2020). Similar to cerebellar granule cells, the axons of around 2000 KCs per hemisphere project as thick bundles of parallel fibers to form the backbone of the MB. The axon bundles form three main lobes, α/α', β/β', and γ (Crittenden et al. 1998). These three lobes form the characteristic MB structure with vertical and horizontal lobes (Fig. 1b). α/α' form the vertical lobe structures, and β/β' and γ together form horizontal lobes (Aso et al. 2014). The cholinergic KC somas form the calyx of the MB, and their dendrites receive sensory inputs predominantly from olfactory PNs (Barnstedt et al. 2016; Vosshall and Stocker 2007; Wilson 2013) and additional inputs from gustatory, thermal and hygrosensory (Marin et al. 2020), and visual centers (Kirkhart and Scott 2015; Scott 2018; Vogt et al. 2016; Yagi et al. 2016). In fact, the most recent connectomics project on the MB circuit found an unexpected number of visual projections, both direct and indirect, to the MB (Li et al. 2020a, 2020b). Furthermore, although the connections between sensory input neurons, such as the olfactory PNs, and the KCs is in large parts random (Caron et al. 2013; Murthy et al. 2008), there is some structured representation of, for instance, the highly aversive odor Geosmin (Stensmyr et al. 2012), which is different from the structured input of food odor vinegar or pheromones, indicating that the MB contains some priors regarding the sensory representation of highly ethologically relevant stimuli (Li et al. 2020a; Zheng et al. 2018). The KCs convey information to the dendrites of 21 types of MB output neurons (MBONs) which innervate 15 specific compartments along the MB lobes (Aso et al. 2014). Importantly, in the same MB compartments, axons of 20 types of DANs innervate and form synapses with KCs and MBONs (Fig. 2a). DANs innervating these compartments originate from two DAN clusters, namely, PAM and PPL1 with cell bodies close to the MB. Their dendrites project to different areas in the superior protocerebrum, where they receive input from other cells including MBONs (Aso et al. 2014, Li et al. 2020a, Mao and Davis 2009, Otto et al. 2020). These 15 compartments, thus, form parallel functional units in which each of the compartments are innervated by axons of KCs, dendrites of specific MBONs and axons of specific DANs. The non-overlapping axonal innervation of DANs in each compartment likely delineates anatomical and functional boundaries of the MB compartments (Aso et al. 2014). Within and between these compartments of the MB occur the possibly most complex synaptic connections in the entire fly brain (Aso et al. 2014; Li et al. 2020a; Takemura et al. 2017; Zheng et al. 2018). In the simplest connection diagram, the KC axons in each compartment make synaptic connection to the dendrites of the corresponding MBONs (KC > MBON), and compartment-specific DANs innervate these KC synapses in order to modulate the KC-MBON synapse according to state and experience. While this simple model is fundamentally still correct and conceptually useful to understand how dopaminergic modulation in the MB can lead to changes in behavior and learning, new connectomics work thoroughly revised this model (Eichler et al. 2017; Li et al. 2020a; Takemura et al. 2017) (Fig. 2a).

Even though the MB compartments are anatomically and functionally independent, most of these compartments are extensively interconnected to each other as DANs receive recurrent input from KCs (KC > DAN) and MBON (MBON > DAN) (Fig. 2b–d). In addition, axons of the DANs can make synaptic connections to the dendrites of the MBON within the compartments (Takemura eta al. 2017; Li et al. 2020a). However, some of the most complex connections are formed between MBONs to DAN. Here, MBON axons show extensive feedback connections on the dendrites of DANs at the MBON output neuropil, namely, CRE, SMP, SIP, and SLP where DANs receive recurrent inputs from MBON (Aso et al. 2014; Li et al. 2020a). Three main types of direct synaptic connections were observed between MBON and DAN. First, MBONs and DANs of the same compartment form a reciprocal feedback loop (Fig. 2b). Second, MBONs of one compartment feedback to DANs of a different compartment. This connection facilitates cross compartment communication (Fig. 2c). Third, MBONs from the same and different compartments feedback to the DAN (Fig. 2d). This connection architecture provides the basis of within and across compartment communication. These rich recurrent feedbacks form an important backbone of memory formation, expression and update, and in addition modulate state and context-dependent behavior in flies (e.g., Felsenberg et al. 2017, 2018; Ichinose et al. 2015; Pavlowsky 2018; Perisse et al. 2016; Sayin et al. 2019; Zhao et al. 2018).

Remarkably, similar to the MB network, within the striatum, DAN inputs show compartmentalized segregation (Berke 2018; Ito and Doya 2015; Watabe-Uchida and Uchida 2018), and the striatum can be subdivided in dorso-lateral, dorso-medial, and ventral parts (Voorn et al. 2004). DAN projections follow this segregation without any clear-cut borders. DANs from the VTA project mainly to the ventral striatum while DANs from the substantia nigra project mainly to the dorsal regions of the striatum. These compartments are also functionally distinct. In a very simplified model and similar to the simplified view of DANs innervating the MB (see below), the ventral striatum is involved in reward processing and motivational control, while the dorsal part of the striatum has been associated with motor control, motor and habit learning. It is indeed known since the first studies on Parkinson’s disease that the loss of dopamine innervation in the dorsal striatum causes movement deficits. Note that this reduction of movement has also been viewed as a reduction of motivation, implicitly (Mazzoni et al. 2007).

## DANs as encoders of state, context and experience

Dopamine has been implicated in an overwhelming number of different behaviors in the different models (Scaplen and Kaun 2016). Earlier theoretical models and studies in primates pinpointed a role of dopamine in reward prediction error encoding (Schultz 2015, 2016; Schultz et al. 1997). Newer behavioral studies in rodents and insects have challenged this view and instead suggest that DANs are a highly heterogenous class of neurons that respond, at different time scales, to many types of biological signals including internal, sensory, behavioral state, motivation and punishment (Berke 2018). As a result, a clear or simple answer for “what is dopamine doing?” has not and might never be found. Nevertheless, in our view, one of the most important conclusions of many years of dopamine research is that the, arguably heterogeneous role, of this neuromodulator appears to be conserved across species of very different body plans and living conditions. This remains true for the involvement of specific molecules such as specific types of dopamine receptors to the physiological responses and behavioral functions.

**Fig. 3:**
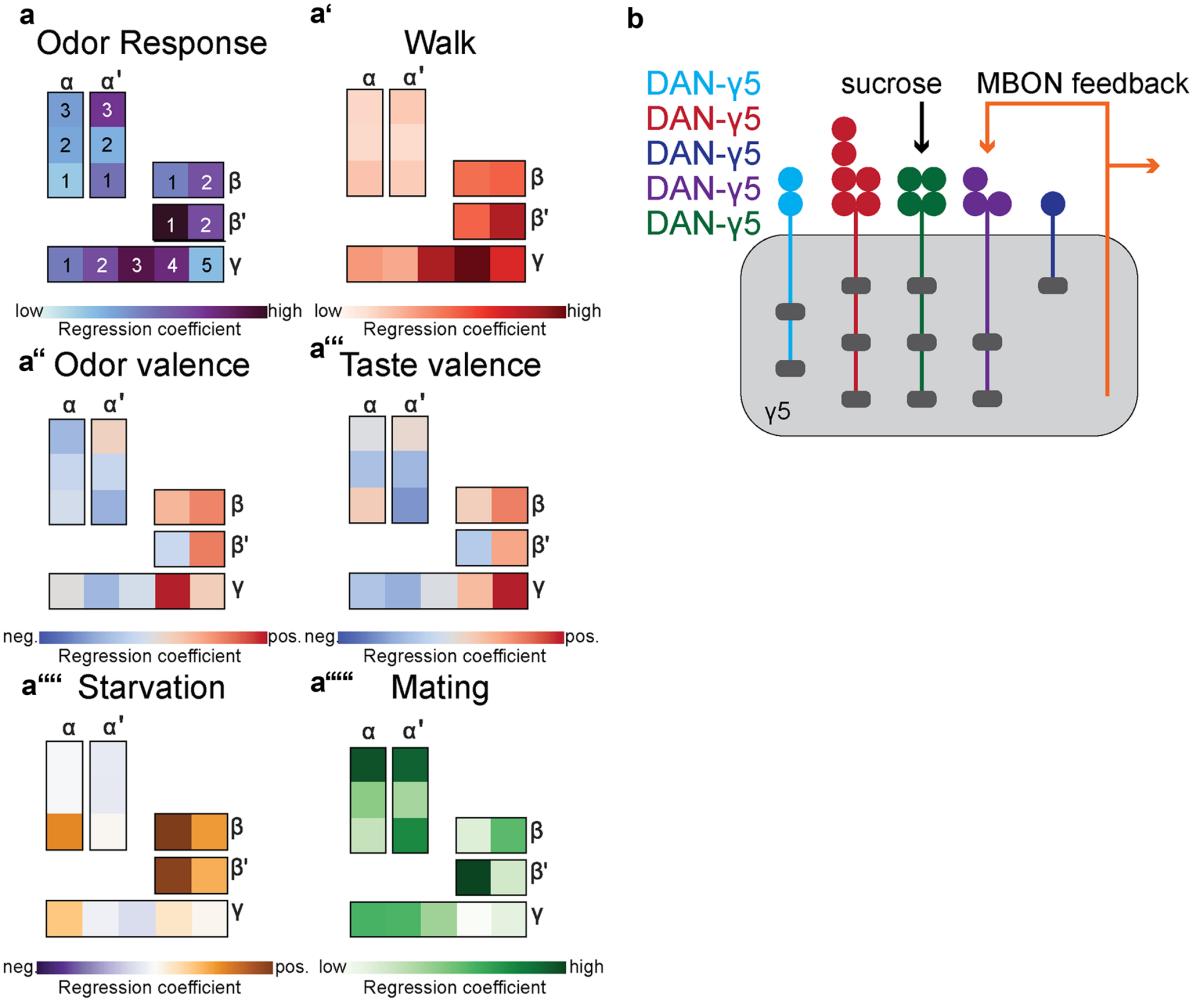
(a-a’’’’’) Dopaminergic neurons encode different types of information (a–a’’’’’) In vivo population calcium imaging suggests that dopaminergic neurons (DAN) encode valence (a’’–a’’’), presence of sensory stimulus (a), metabolic (a’’’’), and reproductive states (a’’’’’) as well as movement (a’) in different and overlapping mushroom body (MB) compartments. Colors indicate strength of regression coefficient from lower or negative (lighter colors) to higher or positive (darker colors) in all panels except panels ‘odor valence’ (a’’) and ‘taste valence’ (a’’’). Here, blue colors indicate correlation with negative valence, where red colors indicate correlation of activity with positive odors. In the starvation panel (a’’’’), blue indicates negative correlation. Note that valence representation is very similar between two sensory modalities (odor: negative vs. positive odors; taste: quinine vs. sucrose). See Siju et al. (2020) for details. (**b**) Simplified schematic showing that the MB γ5 compartment is innervated by five different dopaminergic neuron types. These DANs receive different synaptic inputs and feedback by MB output neurons (MBON). Adapted from Otto et al. (2020)

The use of a large array of interdisciplinary approaches has strongly advanced our comprehension of the diversity of DANs and their biological functions in the *Drosophila* brain. Behavioral analysis has become more ethologically relevant and detailed, capturing the dynamics and different phases of an ongoing behavior rather than simply quantifying the outcome (Branson et al. 2009; Ravbar et al. 2019; Sayin et al. 2019). In vivo imaging and electrophysiology in living and behaving flies has highlighted the diversity of signal type and the importance of timing of DAN activation (Aimon et al. 2019; Handler et al 2019; Siju et al. 2020). DANs respond to various sensory modalities including odor, taste and temperature. These responses are modulated by the internal state (e.g., metabolic and reproductive) and the behavioral state (e.g., moving or not) of the animal (Aimon et al. 2019; Berry et al. 2015; Cohn et al. 2015; Siju et al. 2020; Tomchik 2013; Tsao et al. 2018). Importantly, not all DANs respond the same, and the population activity of DANs across MB compartments can provide more information than individual neuron or compartment responses (Berry et al. 2015; Cohn et al. 2015; Siju et al. 2020). It is worth noting that the population activity of DANs, in line with the somewhat stereotyped odor responses of MBONs (Hige et al. 2015) and the not fully random odor input to KCs (Li et al. 2020a), encodes some information about odor identity (Siju et al. 2020); this might suggest that some odors, or their ethological meaning, could shape responses to other odors as contextual signals during behavior and learning. Consistent with this idea behavioral analysis and imaging provide evidence that DANs contribute to innate valence perception of odors, tastes, and temperature (Siju et al. 2020; Tomchik 2013). Interestingly, while there are some differences between DAN odor and taste responses, valence representation appears to be by and large independent of sensory modality (Fig. 3a–a'''''). Together, many newer and older studies in *Drosophila* come to similar conclusions as work in rodent models: DANs are heterogeneous and appear to flexibly and experience-dependently encode much of the relevant information animals need not only to learn but also to modify ongoing behavior and make appropriate decisions.

An intriguing question is which neurons convey sensory and state-related information to DANs? And how can such input be used in a timing-dependent manner such that the time of DAN activity and presumably dopamine release relative to the presence of a sensory experience (e.g., before or after odor presentation) can determine whether an experience is positive or negative (Handler et al. 2019; Tanimoto et al. 2004). A recent connectomics study showed that DAN compartments might be further divided into sub-compartments as shown in the case of compartment γ5 (Otto et al. 2020). Here, the authors find that five different DANs with distinct dendritic locations and input neurons innervate γ5, possibly explaining the integrative nature of dopamine signaling in a single compartment but also across compartments. This finding thus stresses that even a highly similar type of DAN can process information differentially depending on its sub-compartment architecture (Otto et al. 2020) (Fig. 3b).

With such an interconnected compartmental organizations and wired to receive multimodal internal and external signals (Berry et al. 2015; Cohn et al. 2015; Lewis et al. 2015; Masek and Scott 2010; Owald et al. 2015b; Owald and Waddell 2015; Siju et al. 2020; Vogt et al. 2016), the fly MB represents a bona fide decision-making center in the fly brain that can modulate behaviors at different levels and time frames, making *Drosophila* MB DANs an attractive model to study state-dependent decision-making and adaptive behavior.

### Dopaminergic modulation and metabolic state

One of the most basic needs of an animal to survive is to forage and feed on food to acquire energy and other essential nutrients and maintain vital body functions. A drop in energy and nutrients will trigger foraging and feeding (Lin et al. 2019; Mahishi and Huetteroth 2019), increase arousal and the motivation to seek and remember food and food-related cues (Krashes et al. 2009; Sayin et al. 2019). Hence, foraging and feeding is a motivational state-dependent behavior tightly controlled by internal energy needs, external sensory stimuli, and prior experience (Lin et al. 2019, Tsao et al. 2018). Because of this, animals, including flies, show great adaptability to optimize their energy expenditure during food search, which in nature can be a long-lasting and dangerous process. Dopamine plays a key role in modulating this state-dependent decision-making in flies by conveying internal metabolic state, sensory value of food and reinforcement signals to maintain or change a behavior (Krashes et al. 2009; Lin et al. 2019; Sayin et al. 2019, Tsao et al.  2018).

Flies predominantly use their olfactory and gustatory senses for finding and evaluating food. Not all food cues are equally positive and some are even aversive. For instance, walking fruit flies show innate avoidance behavior toward CO_2_ (Suh et al. 2007). However, at the same time, flies feed on fruits which also produce CO_2_ during ripening and fermentation. Hungry flies overcome their aversion by recruiting pathways in the MB of a specific compartment (β2/β'2a), which is innervated by PAM DANs (Bracker et al. 2013; Lewis et al. 2015). These PAM DANs appear to transmit the valence of a co-incident positive food odor to the MB in a metabolic state-dependent manner, thereby suppressing the CO_2_ response of the MBONs that drive CO_2_ aversion (Lewis et al. 2015). This work indicated that flies use their MB not only for learning about the future, but also to modulate their behavior instantaneously in a state-dependent manner. Metabolic state is, at least in part, communicated to the MB in the form of different neuropeptides, neurotransmitters or hormones via DANs. Extensive genetic screening revealed that DANs in compartments α3, β2/β'2a, α'2 α2, γ3, γ2/α'1, and γ1pedc receive different feeding state-related signals, which modulate the corresponding MBONs and ultimately foraging behavior (Tsao et al. 2018, Yamagata et al. 2016). This modulation is also dependent on a dopamine receptor, Damb/Dop1R2, expressed in KCs and MBONs (Tsao et al. 2018). In vivo population imaging of MB DANs recently reported additional metabolic state-sensitive DAN sub-types (β1 and β'1) (Siju et al. 2020). Together, these findings show that feeding state modulates dopaminergic signaling in several MB compartments and thereby adapts feeding related behavior according to the animal’s need.

An important aspect of decision-making is accurately recalling past experiences that are stored as memory. Dopamine, as extensively reviewed by Fiala et al. (same issue), plays an important role in learning and memory in flies (Cognigni et al. 2018; Kaun and Rothenfluh 2017; Modi et al. 2020). In hungry flies, DANs provide reinforcing signals to form and express appetitive memories of food-associated odors (Burke et al. 2012; Krashes et al. 2009; Liu et al. 2012a; Musso et al. 2015; Placais et al. 2012). Such reinforcing signals of sweet taste to form short-term memory are relayed by PAM β'2 and γ4 DANs, whereas reinforcing signals for long term memory formation of the nutritional value of sugar are provided by DANs projecting to γ5, β1, β2, α1, and γ1pedc (Huetteroth et al. 2015; Musso et al. 2015; Pavlowsky et al. 2018; Placais et al. 2017; Siju et al. 2020; Yamagata et al. 2015). Not only formation but also memory expression are directly dependent on the feeding state of the fly. In fed flies, PPL1-γ1pedc DANs inhibit their corresponding MB compartment so that no sugar memory is expressed (Krashes et al. 2009). In a hungry animal, these inhibitory signals are suppressed by the action of neuropeptide dNPF, which is released in response to starvation (Krashes et al. 2009).

Interestingly, hunger modulates not only the strength but also the way a DAN will respond to a stimulus. Slow spontaneous activity of PPL1-γ1pedc DANs together with PPL1-γ2α'1 DANs underpin aversive long-term memory (LTM) formation in fed flies (Placais et al. 2012). By contrast, when starved, the tonic activity of these DANs is drastically reduced blocking the formation of aversive LTM. Thus, in an energy demanding situation like starvation, forming aversive LTM is possibly too costly for the animal such that DANs effectively enable flies to save energy in a feeding-state dependent manner (Hirano et al. 2013; Plaçais and Preat 2013).

As mentioned above, finding food is, for most animals, a dangerous, costly and long-lasting affair, which is, on top of it, not always successful. How do hungry animals decide whether to continue or abandon the search for food? In a recent publication, (Sayin et al. 2019) showed that this strong motivation to maintain and even increase efforts in food search behavior when unrewarded is controlled by subsets of MB DANs (Sayin et al. 2019). Inactivation of subsets of DANs present in the PPL1 and PPL2 clusters led to a strong decrease in odor tracking behavior on a spherical treadmill. Further, they found that Dop1R2 receptor signaling in α/β KCs mediates and modulates this motivated searching behavior (Sayin et al. 2019). Intriguingly, the mechanism and neural circuit driving the maintenance and increase of food-seeking behavior of hungry flies over repeated trials closely resembles the underpinning mechanism of olfactory memory formation. This led to the hypothesis that dopamine enables working memory to update sensory and goal representation during ongoing behavior (Preat and Placais 2019).

Dopamine also modulates behavior once the hungry animal has found the food source. Feeding starts with extending the proboscis, tasting the food, and is followed by food ingestion. The decision to extend the proboscis and initiate the feeding process is controlled by a single DAN (Marella et al. 2012). This DAN, located in the SEZ and known as TH-VUM (tyrosine hydroxylase ventral unilateral medial) neuron, directly influence the proboscis extension response in a metabolic state-dependent manner such that proboscis extension increases when the flies are hungry and the TH-VUM neurons are active (Inagaki et al. 2012; Marella et al. 2012). Moreover, dopamine can modulate sugar sensitivity of SEZ neurons in a starvation-dependent manner, without affecting bitter sensitivity (Inagaki et al. 2012, Inagaki et al. 2014; Marella et al. 2012). An increased sugar sensitivity during foraging reduces the animal’s aversion of food that is not rich in sugar.

Together, these studies show that dopamine can exert metabolic state-dependent modulation at multiple levels in the fly nervous system, from a very early stage of sensory processing up to higher brain centers to enable appropriate foraging and feeding decisions.

### Need for specific nutrients are conveyed via dopamine

In particular, during development, flies need various nutrients in addition to just energy, and the need for these nutrients changes with developmental stage. The mechanism by which flies sense and ingest micro and macro nutrients other than sugars are not well understood. However, a recent study showed a clear link to dopaminergic modulation in protein-craving flies (Liu et al. 2017). When male flies are low on protein, especially after mating, their preference for normal food containing sugar is switched to a more protein-rich food. This state-dependent decision to eat protein rich food is regulated by a specific set of DANs in the PPM2 cluster, which are connected to wedge-neurons. Artificially activating these DANs increased the preference for protein rich food compared to sugar even in protein sated males (Liu et al. 2017).

This study provides evidence that dopamine can help animals to make very specific feeding choices by adjusting reward value circuits to current internal state.

### Dopamine influences the choice of thirsty flies

Drastic changes in environmental temperature, humidity, body osmolality, and ingestion of certain foods can cause animals to feel thirsty. Since water is crucial for the body, this need is monitored by several modulatory factors and often elicits strong behavioral responses in animals. Thirsty flies detect the presence of moist air through hygroreceptors in their third antennal segment; by contrast, hygroreceptors in the arista are involved in sensing moist air to avoid further water exposure when the animal is sufficiently hydrated (Ji and Zhu 2015; Liu et al. 2007). Once in contact with water, the gustatory system through the osmosensitive channel PPK28 initiates water imbibing (Cameron et al. 2010; Chen et al. 2010). Importantly, water drinking appears to be a rewarding process only for water deprived flies (Lin et al. 2014; Liu et al. 2007; Shyu et al. 2017). Since water is crucial for survival, flies memorize sensory cues associated with water with the help of dopamine: water information, similar to the presence of an attractive odorant (Lewis et al. 2015), is conveyed by β'2 innervating DANs (Lin et al. 2014). Moreover, a recent study reported that Dop1R1 mutant flies consumed less water compared to wild-type flies (Lau et al. 2017). This study further showed that the palpability of water tasting increases because of dopamine release in thirsty animals (Lau 2017). Through DANs, flies can also form short-term memory (STM) and LTM to cues associated with water. LTM for water is reinforced by PAM β'1 neurons and Dumb/Dop1R1 in α'/β' KC (Shyu et al. 2017). On the other hand, DANs innervating the γ4 compartment are required for inducing STM of water (Lin et al. 2014; Shyu et al. 2017). It is interesting to note that, similar to sugar memory formation, STM and LTM for water cues are mediated by two different subsets of DANs, possibly giving the animal higher flexibility in its choices.

Thus, while animals for obvious reasons benefit from remembering where to find water, it is equally important to balance their intake of water and nutrients. In a recent study, Senapati et al. (2019) identified that DANs are involved in prioritizing the expression of water or sugar memory when thirst and hunger arise at the same time (Senapati et al. 2019). Essentially, flies choose whether to drink water or feed on sugar according to two competing needs. Leucokinin, a neuropeptide, which is released in thirsty flies, specifically inhibits two types of DAN subsets, PAM-β'2a and PPL1-γ2 α'1, thereby promoting water memory expression. Leucokinin can also promote sugar memory in flies through the activation of PAM-β'2mp DANs. Surprisingly, when hunger and thirst occur simultaneously, hunger wins. This is because water memory is somehow neutralized by hunger-promoting signals such as neuropeptide dNPF and serotonin in “water” DANs (Senapati et al. 2019). Hence, dopamine enables flies to dynamically prioritize a choice depending on their need by promoting or inhibiting behaviors in a need-dependent manner.

### Dopamine regulates reproductive success

One of the most important aspects of animal life is reproduction to ensure gene flow and evolutionary success. As animals go through different reproductive states, they exhibit changes in their physiology and behavior. This plasticity is controlled and coordinated by different neural circuits at different levels in the reproductive machinery (Auer and Benton 2016). In addition to some other neuromodulators, dopamine plays a prominent role in controlling reproductive state behaviors such as courtship, mating or egg laying (Sayin et al. 2018). Courting and mating involve elaborate sequential rituals in flies (Aranha and Vasconcelos 2018). Moreover, courtship and mating are intertwined behaviors: only successful courting will lead to mating (Zhang et al. 2018). Flies recognize their appropriate mating partners using different sensory cues such as visual, acoustic, olfactory, gustatory, and tactile. However, communication via the olfactory system plays a major role in reproductive behaviors (Billeter and Wolfner 2018). Some of the animal’s own cuticular hydrocarbons serve as sex pheromones, and among them, cis-vacenyl acetate (cVA) is the most prominent and most studied (Brieger and Butterworth 1970; Ferveur 2005; Ha and Smith 2006; Keleman et al. 2012; Kohl et al. 2015; Sengupta and Smith 2014).

When to court and when not to court is an important decision for animals, as in some cases, they have to prioritize more pressing needs, for instance, feeding or escaping from predators (Zhang et al 2018). In male flies, the decision to engage or disengage in courting is centrally controlled by male specific command neurons known as P1 (Kimura et al. 2008). This class of neurons integrates motivational control for courtship from the aSP4 DAN of the PAL DAN cluster and courtship related sensory cues, and projects it to a higher brain area called SMPa (Zhang et al. 2018, 2016). A current model suggests that increased dopamine release sustains the motivational level of courtship, which decreases once mating needs are satisfied. Direct evidence was obtained by optogenetically activating courtship reporting neurons, which decreased DAN output activity at the level of the SMPa area, indicating reduced release of the dopamine during mating (Zhang et al. 2018, 2016). Similarly, repeated activity of the DAN also reduced sex drive. However, after a few days, the males, due to rising dopamine levels, started to court again. PAL DAN activity itself is modulated by the activity of NPF neurons that also receive courtship state information from the courtship reporting neurons (Zhang et al. 2019). Similarly, dopaminergic modulation is also involved in age-dependent courtship motivation in male flies. Aged male flies show less vigor in courtship and mating compared to reproductively active younger males, which appears to depend on the activity of a subset of PPL2ab DANs (Kuo et al. 2015).

Dopamine, however, does not only regulate ongoing courting or mating activity. Male flies can with the help of DANs remember their previous sexual experiences and the outcome. When a naïve male fly tries to court an already mated female, the courting will usually not be successful as the female will reject the male. Mated females, importantly, smell different, because they emit male cVA transferred from their previous sex partner. MB γ-compartment DANs and Dop1R1 are important for this “courtship learning” (Keleman et al. 2012). When a male fly experiences rejection by the female, its response toward cVA markedly increases. The dopamine released by PAMγ5/aSP13 DANs activates the γ5 MBON in a recurrent excitatory loop, and thereby determines the duration and strength of the negative courtship memory depending on the number of courtship attempts by the male. Moreover, in another report investigating courtship learning, Montague and Baker showed that courtship memory also involves α/β KCs, in addition to the γ lobes (Montague and Baker 2016).

Not only males but also female flies undergo changes in their perception and behavior upon mating. For instance, food and odor preferences, behavioral priorities, and mating receptivity are different in a mated female as compared to a virgin (Hussain et al. 2016a, 2016b; Liu and Kubli 2003; Walker et al. 2015). Siju et al. (2020) recently showed through in vivo calcium imaging that specific DANs are modulated depending on the mating state of the female. DANs innervating β'1 and α3 compartments of the MB showed higher responses to the sex pheromone cVA in mated females compared to virgin females (Siju et al. 2020). A higher sensitivity to cVA in the DANs after mating provides a hint that the MB could enable mated female flies to express several postmating related behaviors. For instance, since cVA is a pheromone transferred to females during mating, conspecific females might use this cue to locate other mated females to select a substrate that is already used for oviposition by other females (Dumenil et al. 2016; Sarin and Dukas 2009). Alternatively, cVA might suppress a mated female’s attraction to males.

The next step after mating is egg laying or oviposition. Since flies do not take care of their offspring after egg laying, it is crucial for female flies to choose the right substrate for ovipositioning and increase the survival of their offspring. Flies use several sensory cues to locate suitable oviposition substrates (Azanchi et al. 2013; Markow and O’Grady 2008) and to orchestrate behaviors during the egg-laying process (Aranha and Vasconcelos 2018). Females choose egg-laying substrates according to a value-based decision-making process (Yang et al. 2008). In a follow-up study, Yang et al. (2015) showed that a specific subset of DANs is important for an egg-laying preference on sucrose-containing substrates (Yang et al. 2015). These authors showed that increased activity of PAL and PPL2 DANs increased the preference of sucrose-rich substrate for egg laying (Yang et al. 2015). However, they did not find any direct involvement of the MB in this decision-making process (Yang et al. 2015). By contrast, another study by Azanchi et al. implicated the MB and showed that PAM and PPM3 DANs enhance oviposition preference for ethanol-containing substrates, while PPL1 DANs do the opposite. These opposing effects of dopamine may help in deciding whether to deposit eggs on fruits containing low or high concentrations of ethanol: as low concentration is a preferred choice as high alcohol concentration can be detrimental (Azanchi et al. 2013; Yang et al. 2008).

In spite of this clear evidence for role of dopamine in reproductive state-dependent behavior, it is not known which upstream circuits convey state and value signals to the DANs.

### Social behavior is tuned by dopaminergic neurons

Aggression is a state-dependent behavior, and most animals show aggressive behaviors in the context of resources, mates, and predators that often culminate into fight (Anderson 2016; Asahina 2017). Moreover, internal states can influence and enhance aggressive behaviors (Anderson 2016). The decision to fight or not to fight is important as aggressive behavior can further deplete energy levels or even lead to death (Hoopfer 2016). Still, hungry, but not fed, flies in the context of limited food resources show aggressive behaviors toward conspecifics (Lim et al. 2014). Similarly, male flies show competitive and aggressive behavior when in the mood for mating (Bath et al. 2017; Chapman and Wolfner 2017). Interestingly, aggressive and courtship behaviors are both elicited by cVA (Wang and Anderson 2010).

Some aspects of these behaviors, or when to express them, are under dopaminergic regulation. For example, two sets of DANs found in both T1 and PPM3 clusters are involved in modulation of aggression in flies (Alekseyenko et al. 2013). Interestingly, both inactivation and activation of these two sets of DANs promote aggression; however, the mechanism involved is not yet fully understood (Alekseyenko et al. 2013). More recently, Kim et al. provided strong evidence that repeated aggressive fights that were lost led to the formation a long-lasting aversive memory in males through a PPL1-γ1pedc- and corresponding MBON-dependent modulation (Kim et al. 2018). This aversive memory could be useful in situations where flies have to prioritize foraging or mating over fighting.

### Environmental temperature preference relies on dopamine

Drosophila flies are considered to be tropical in origin coming from east equatorial Africa (Hansson and Stensmyr 2011). Over the course of their cosmopolitan spreading across the world, flies adapted to different temperature ranges from cold to warm (Hansson and Stensmyr 2011; Trotta et al. 2006). Flies are poikilothermic animals, unable to adjust their internal body temperature independent of environmental temperature. If and when environmental temperature changes, flies need to adjust their body temperature by moving to an appropriate environment. Hence, flies constantly evaluate changes in their environmental temperature and show behavioral reactions to the temperature changes (Barbagallo and Garrity 2015; Bellemer 2015; Dillon et al. 2009; Nevo et al. 1998).

Flies sense external temperature through three thermosensory sensilla in the arista. Each of these sensilla houses one cold activated and one hot activated cell (Alpert et al. 2020; Gallio et al. 2011). In addition, flies also have internal thermal sensors in the brain known as anterior cells expressing the dTrpA1 receptor (Hamada et al. 2008). Since changes in temperature need to be evaluated and integrated with context and internal state, it would not be surprising if temperature homeostasis were subject to neuromodulation (Lubawy et al. 2020). Supporting this idea, several studies showed that DANs are involved in temperature preference behavior. More specifically, MB DANs receive temperature stimuli, and silencing the output of a large fraction of DANs changed preferred temperature behavior in flies (Bang et al. 2011; Hong et al. 2008; Tomchik 2013). These behavioral data are corroborated by functional imaging of DANs. DANs in the vertical lobe, especially PPL1-α3/α'3, PPL1-α2α'2, PPL1-γ2α'1, PPL1-γ1pedc respond to decreases in temperature (Tomchik 2013). Interestingly, another study showed that PAM-β'2 and PAM-β2 are involved in modulating cold temperature avoidance in flies (Shih et al. 2015). By contrast, two independent studies showed that DANs are not involved in reflexive responses to high temperature (Galili et al. 2014; Tomchik 2013). Nevertheless, similar to cold temperature avoidance, DANs act as reinforcers in high temperature avoidance learning. (Galili et al. 2014) reported that a larger population of DANs labeled genetically by a TH-D’-GAL4 transgene (Liu et al. 2012b) convey painful temperature very similar to electric shock during aversive olfactory memory formation (Galili et al. 2014). These DANs innervate mostly the vertical lobe and, surprisingly, overlap with the DANs involved in the avoidance of cold temperatures (Galili et al. 2014; Tomchik 2013). How exactly DANs and the MB allow flies to evaluate and chose appropriate temperature environments, and whether these neurons integrate additional factors such as metabolic state or history of temperature changes remains an interesting and open question.

### DANs respond to movement

Unless resting, most of the time flies are on the move to find food, mates, oviposition sites, or to escape from predators or harsh environment. It implies that a fly’s movement is influenced by its motivational state, external sensory environments, and behavioral output. Although dopamine activity increases strongly in many regions over the brain during walk without any sensory stimulus (Aimon et al. 2019), recent works have mostly focused on MB DANs (Aimon et al. 2019; Berry et al. 2015; Cohn et al. 2015). For example, the activity of DANs innervating γ2α'1 and γ3 compartments was correlated with walking movement on a ball (Berry et al. 2015; Cohn et al. 2015). Expanding on these results, another study, using population imaging of DANs, showed that walking is correlated with increased activity of several DAN compartments in the horizontal MB lobes. In particular, β1, β2, β'2, and γ3-5 DAN compartments showed strong activity during walking movement (Siju et al. 2020). Interestingly, modulation of DAN activity was similar for movement and internal state such as hunger, mating, and sensory valance (Siju et al. 2020).

Why many DANs show a higher activity during movement is an intriguing question. One possibility is that heightened activity during movement may enable these DANs to get in a more excitable state in which even a little external sensory cue will activate them to modulate behavioral decisions. The behavioral state (e.g., moving vs. grooming) could be important information in itself for further decisions, and dopamine could carry operant learning signals (Sun et al. 2020). Studies looking at the precise timing of activation as well as what aspect of the behavior is coded (for example whether DANs represent just walking or not, the speed of the walk or even the trajectory) will help answer this question.

As said before, this is reminiscent to what happens in the mammalian brain. Indeed, it is known from Parkinson’s disease that dopamine is involved in motor control, and artificial stimulation of overall DANs also promoted movement initiation (da Silva et al. 2018; Howe and Dombeck 2016). Direct observation showed that DANs fire phasically before spontaneous movement initiation non-triggered by an external stimulus (Coddington and Dudman 2018; Dodson et al. 2016; Howe and Dombeck 2016; Syed et al. 2016), and many DANs are also active during the movement (Engelhard et al. 2019; Howe and Dombeck 2016). However, in flies, so far, all available data point to that DAN activity is either synchronized to movement or DAN activation initiates movement. For instance, artificial activation of DANs triggers altered locomotion (Lima and Miesenböck 2005). Therefore, whether DANs primarily control movement, respond to movement or both is difficult to answer with determination at this point. Being able to manipulate DAN activity precisely in time and space has not only substantiated dopamine’s function in learning and change of future behavior, it has also provided evidence for a role of DANs in changing ongoing behavior in insects (Berry et al. 2012; Cohn et al. 2015; Lewis et al. 2015, Tsao et al. 2018) and rodents (Saunders et al. 2018). It is possible that this is, at least in part, explained by the reinforcing action of movement-modulated DANs (Coddington and Dudman 2019).

### Sleep is regulated by dopamine

Sustained activity-dependent behaviors and arousal mean that animals stay in a continuous wakeful state for a prolonged time. This continuous wake state puts constraints on the physical and physiological state of the animal and eventually forces them to rest or sleep. Although sleep has been observed in almost all animals, how sleep is initiated, maintained, and modulated remains poorly understood. In flies, a rest period or immobility of 5 min or more with a reduced response to sensory stimuli is termed as sleep (Hendricks et al. 2000). Sleep affects several state-dependent behaviors such as feeding, mating, as well as learning and memory in flies (Donlea et al. 2014). Sleep is also a motivated behavior and both external sensory cues and internal signals can affect sleep (Nall and Sehgal 2014). In line with this, pioneering studies showed that dopamine is an important molecule controlling sleep in flies. Kume et al. identified a dopamine transporter (DAT) gene mutant named *fumin* and showed that these mutant flies sleep less compared to wild type flies (Kume et al. 2005). This sleep loss was attributed to increased amount of dopamine in synaptic junctions that was not cleared because of the DAT mutation (Kume et al. 2005). In support of this finding, another study showed that an increased concentration of dopamine in flies reduced sleep and promoted wakefulness and activity (Andretic et al. 2005).

Similar to mammals, multiple sleep regulating centers have been found in the fly brain (Dissel 2020). The dorsal fan shaped body (dFB) and MB are thought to be the main sleep centers in flies. It has been found that activation of the dFB neurons promoted sleep in flies (Donlea et al. 2018). By contrast, DANs projecting to dFB disrupt sleep and increase wakefulness. Two independent studies showed that DANs of PPM3 and PPL1 clusters project to dFB and activation of these DANs reduces sleep and increases wakefulness. However, when silencing these DANs, sleep is increased (Liu e al. 2012b; Ueno et al. 2012). Further studies by Pimentel et al. showed that dopamine released by these DANs onto the dFB electrically silences dFB neuron excitability to suppress sleep and increase wakefulness; this silencing is mediated by Dop1R2 receptors present on the dFB cells (Pimentel et al. 2016). The dFB is a part of the CC which also comprises other neuropils such as ventral fan shaped body (vFB), protocerbarl bridge (PB), and ellipsoidal body. Interestingly, some of the recent findings have also implicated vFB and PB in a dopamine-mediated control of sleep (Dag et al. 2019; Duhart et al. 2020). DANs projecting to PB (DA-PB) neurons are involved in sleep modulation in a nutrient-dependent manner, where yeast-deprived male flies show increased wake activity and reduced sleep (Duhart et al. 2020). This finding is in line with a previous observation that starvation reduces sleep in flies (Keene et al. 2010), and together stress that dopaminergic neurons integrate different states and may compare their respective urgency.

Another brain center in the fly that has been implicated in sleep regulation is the MB (Joiner et al. 2006; Pitman et al. 2006). However, a direct link to MB projecting DANs and their involvement in sleep and wake regulation was provided only recently (Berry et al. 2015; Sitaraman, et al., 2015b). Artificial activation of PPL1 and PAM DANs projecting to distinct MB compartments decreased sleep and silencing the activity of PAM DANs, especially PAM-γ5, increased sleep (Sitaraman et al. 2015a). These findings confirm that MB DANs have wake-promoting functions and project primarily to specific wake-promoting MBON compartments such as γ4, γ5, and β'2 (Sitaraman et al. 2015b).

Sleep has been shown to influence learning and memory in flies. Seugnet et al. showed that sleep deprivation impairs learning and the concentration of dopamine in the head was increased in sleep-deprived flies, while Dop1R1 receptor transcripts were downregulated (Seugnet et al. 2009). Sleep is equally important for memory formation and consolidation in flies. In a recent study, Dag et al. showed that sleep and DAN signaling is important to consolidate memory in male flies that have experienced courtship rejection from already mated females (Dag et al. 2019). The PAMγ5/aSP13 DAN that is involved in memory formation of this negative experience is further activated during sleep by sleep promoting vFB neurons, indicating that continued DAN activity during sleep strengthens memory (Dag et al. 2019). Interestingly, another study showed that the same PAM-γ5 when activated increases wakefulness (Sitaraman et al. 2015a). One plausible explanation for this discrepancy is that wake-promoting DANs and vFB-activated DANs may be innervating different sub-compartments of the γ5 compartment of the MB (Dag et al. 2019, Otto et al. 2020).

Although the functional significance of sleep remains unclear (Geissmann et al. 2019), from the above account, it is evident that dopamine plays an important role in sleep regulation in flies. Given that sleep and other behaviors are interlinked, dopamine could integrate information from different states and sensory cues to balance different needs.

## Conclusions and future directions

The powerful combination of genetic tools, connectomics, in vivo physiology, and quantitative behavioral analysis in different model systems including *Drosophila* has led to a significant shift in our view and understanding of dopamine and dopaminergic neurons over the last years. DANs are highly heterogeneous and involved in nearly all processes that increase the animal’s success at survival and reproduction.

How DANs in the fly obtain information about valence, state, or movement is among the open questions that will likely be answered at least in part by connectomics (Li et al. 2020a, Otto et al. 2020). Moreover, DANs not only modulate, they are targets of neuromodulation themselves. While receptors for such neuromodulators have been found in insect DANs, their source is frequently not known. Furthermore, are receptor-expressing neurons responding to local, neuronal, or rather to systemic signals released into circulation by other organs, or both? The high degree of recurrent connections, including long-range connections, to and from DANs in the fly brain, represents an ideal mechanism to implement such an immediate, action or need related feedback from other neurons to DANs.

At this point, we know surprisingly little about what happens in dopamine-receiving cells. While often the receptor involved in a particular behavior has been identified, the nature and timing of signal and subsequent activation remain elusive. And finally, it is well known that DANs can co-release other neurotransmitters such as GABA or glutamate (Zell et al. 2020). How is a coordinated or perhaps even more difficult to explain, alternative release of dopamine and another transmitter controlled? And do they affect the same downstream synapses?

While these points certainly do not exhaust the list of open questions, we believe that these are among the most pressing, which at the same time have answers in reach.
